# Lectures on the Blood, and on the Anatomy, Physiology, and Surgical Pathology of the Vascular System of the Human Body, Delivered before the Royal College of Surgeons of London, in the Summer of the Year 1819

**Published:** 1820-01-01

**Authors:** 


*Lectures on the Blood, and on the Anatomy, Physiology, and Surgical Pathology of the Vascular System of the Human Body, delivered before the Royal College of Surgeons of London, in the Summer of the Year* 1819. By JAMES WILSON, F. R. S. Professor of Anatomy and Surgery to the College, and Lecturer on Anatomy and Surgery in the Hunterian School in Great Windmill Street. One volume Octavo, 429 Pages. London, 1819.

Mr. WILSON has long been known as one of the most accurate anatomists of the present day, and as a successful teacher of that fundamental branch of medical science, in the venerable school of the celebrated Hunters. His Lectures, therefore, may be considered as reflecting, *veluti in speculo,* our present state of knowledge on the interesting subjects which he has chosen for delineation before the college. He has not travelled beyond the boundary of solid and well ascertained facts, to amuse his audience with dazzling theories, metaphysical subtleties, or hypothetical speculations. He has written a book which the young man may study, with the view of acquiring knowledge—the old man peruse for the sake of refreshing his memory, and becoming acquainted with the progression of the science—and which all ranks may quote and refer to, as a standard record, documentary of our present knowledge respecting the topics embraced in the work.

It is needless to state, that a book of this kind, so nearly bordering on the elementary, and itself written in a style of such analytical concentration, that scarcely a word could be struck out without injuring the sense, is totally incapable of analysis. We shall not, therefore, attempt what is beyond our power to perform; but merely lay before the reader one or two short specimens of the matter and manner, recommending the work, in the character above described, as well deserving the patronage of the practical surgeon and anatomist. The following passage relative to the treatment of Erysipelas may serve as one.

"If there should be any peculiarity in the constitution likely to act as a predisposing cause of this disease, we would of course direct our attention to the removal of it. As irritation frequently produces it both in the part and in the system, any known irritating cause ought therefore to be removed, or if that cannot be done, should at all events be lessened. If it arises in a part in consequence of a wound, soothing applications to the part, with lenient purges or glysters, will often remove it in a little time; and in a naturally good constitution, where the erysipelas arises from local irritability, particularly in cold climates, the removal of a moderate quantity of blood from the arm, may prove useful. Topical bleeding does not answer; on the contrary, the orifices through which the blood is drawn, will sometimes form very troublesome ulcers, and even gangrene will occasionally arise round them.

"When erysipelas happens in a weak and irritable constitution, a different treatment will be found adviseable; in this case, bleeding and purging should be avoided; but if the patient is costive, clysters should be used. Tonic medicines may be given; and wine, with nourishing food, may be proper. In a true phlegmonous inflammation, bark is found to increase the inflammatory symptoms, and this it certainly will do, whenever the inflammation is kept up from distention; but in erysipelatous inflammation, it rarely, if ever, happens that the inflammation is kept up from distention; it most frequently is kept up from great irritability. Bark, therefore, in these cases, acts from destroying the irritability, and thus takes off the cause continuing the erysipelatous action. The applications which answer the best in removing the affection from the parts, are those of a sedative nature, and it will often prove useful to join to them substances of an astringent power; solutions of opium, alkohol, acetous acid, lime water, and vegetable astringents, may be used separately, or in combination, according to circumstances. Emollient fomentations and poultices are generally improper, for they, like preparations of lead, appear to lessen the power as well as the action of the part.

" When, from a mixture of phlegmonous with erysipelatous inflammation, a thin half-formed pus is deposited, we should endeavour to excite that quantity of inflammation that will prevent it from being diffused. In this state, bark, sulphuric acid, and other tonics have been administered with advantage. A common poultice, having a solution of opium mixed with it, and occasionally a spirituous fomentation with opium, will here be found useful. Should fluid be deposited, openings at the most convenient or dependent part may be made to evacuate it as soon as possible; but the parts in this state will not always bear extensive cutting, nor will they afterwards bear any irritating application. In the species of the disease styled erysipelas phlegmonoides, which very often attacks the legs of seamen, and is often attended with most alarming consequences from the great quantity of matter effused between the integuments and muscles, Mr. A. Copland Hutchison has found that making several free incisions with the scalpel on the inflamed surface, in a longitudinal direction, as early as possible, and before any secretion of matter had taken place, has been attended with very beneficial effects. This incision, he recommends, should be carried through the skin and adipose membrane to the muscles." 342.

The following curious pathological fact is worthy of record.

" I have met with only one instance of true aneurism affecting any of the branches of the aorta which are distributed to the abdominal viscera. In the year 1809, on inspecting the body of a clergyman, in the presence of the late Sir Walter Farquhar, a tumour, very much resembling the heart in colour, shape, and size, appeared to hang down from the under surface of the left lobe of the liver; the lobe itself appeared not more than one sixth part of its usual magnitude. When the tumour was opened and carefully inspected, it appeared to have been formed by the left branch of the hepatic artery having become very much enlarged and aneurismal. It had burst, and the blood which had escaped was found in an imperfect cyst, partly in a fluid, and partly in a coagulated state, forming a large proportion of the tumour. Where the tumour joined the liver, a large cavity was formed in it, which contained a mass of coagulable lymph in a solid state, and about five ounces of a brownish fluid; I could not, however, trace any communication between the enlarged part of the artery and this cavity, although it appeared very like that of another aneurism. The preparation is preserved in the Museum in Windmill Street.

The foregoing extracts are not presented as specimens of the work, but of the matter of which it is composed. Who would offer a single brick as a specimen of the architecture of a solemn temple?

